# *De novo* transcriptome analysis of rose-scented geranium provides insights into the metabolic specificity of terpene and tartaric acid biosynthesis

**DOI:** 10.1186/s12864-016-3437-0

**Published:** 2017-01-13

**Authors:** Lokesh K. Narnoliya, Girija Kaushal, Sudhir P. Singh, Rajender S. Sangwan

**Affiliations:** Center of Innovative and Applied Bioprocessing (A National Institute under the Department of Biotechnology, Govt. of India), S.A.S. Nagar, Mohali, Punjab India

**Keywords:** Rose-scented geranium, *Pelargonium* sp. cv. Bourbon, *De novo* transcriptome, Terpene, Tartaric acid, Ascorbic acid, Anacardic acid

## Abstract

**Background:**

Rose-scented geranium (*Pelargonium* sp.) is a perennial herb that produces a high value essential oil of fragrant significance due to the characteristic compositional blend of rose-oxide and acyclic monoterpenoids in foliage. Recently, the plant has also been shown to produce tartaric acid in leaf tissues. Rose-scented geranium represents top-tier cash crop in terms of economic returns and significance of the plant and plant products. However, there has hardly been any study on its metabolism and functional genomics, nor any genomic expression dataset resource is available in public domain. Therefore, to begin the gains in molecular understanding of specialized metabolic pathways of the plant, *de novo* sequencing of rose-scented geranium leaf transcriptome, transcript assembly, annotation, expression profiling as well as their validation were carried out.

**Results:**

*De novo* transcriptome analysis resulted a total of 78,943 unique contigs (average length: 623 bp, and N50 length: 752 bp) from 15.44 million high quality raw reads. *In silico* functional annotation led to the identification of several putative genes representing terpene, ascorbic acid and tartaric acid biosynthetic pathways, hormone metabolism, and transcription factors. Additionally, a total of 6,040 simple sequence repeat (SSR) motifs were identified in 6.8% of the expressed transcripts. The highest frequency of SSR was of tri-nucleotides (50%). Further, transcriptome assembly was validated for randomly selected putative genes by standard PCR-based approach. *In silico* expression profile of assembled contigs were validated by real-time PCR analysis of selected transcripts.

**Conclusion:**

Being the first report on transcriptome analysis of rose-scented geranium the data sets and the leads and directions reflected in this investigation will serve as a foundation for pursuing and understanding molecular aspects of its biology, and specialized metabolic pathways, metabolic engineering, genetic diversity as well as molecular breeding.

**Electronic supplementary material:**

The online version of this article (doi:10.1186/s12864-016-3437-0) contains supplementary material, which is available to authorized users.

## Background

Rose-scented geranium (*Pelargonium* sp.) is a perennial aromatic and medicinal herb of family Geraniaceae. The genus *Pelargonium* contains about 750 species growing in temperate and subtropical climate [1]. Most of them were indigenous to South Africa, introduced in Europe during 17^th^ century, and subsequently spread all over the world [2, 3]. Aroma possessing species of geranium, such as *P. graveolens* (synonym-*P. roseum*), has a history of folkloric significance. Aerial parts of rose-scented geranium have traditionally been used as insect repellent, perfume and flavouring agents, antimicrobial and aroma-therapeutic herb as well as medicinal plant material of advantage in gastrointestinal disorders, hyperglycemia, and healing [4, 5].

The vegetative and reproductive aerial parts of rose-scented geranium develop numerous epidermal emergences of glandular and non-glandular nature, known as trichomes [6]. The non-glandular trichomes, often unicellular, sometimes bicellular and rarely multicellular, could be physiologically beneficial to plants during temperature regulation, reduction of water loss and, metal tolerance. [7]. Glandular trichomes, the most numerous in leaves, are specialized tissues comprised of a basal stalk and a head of secretory cells that accumulate essential oils [6]. Essential oils are complex volatile compounds, such as terpenes, esters, alcohols, aldehydes, ketones, and phenols, produced in plants as bioactive secondary metabolites, often for ecological adjustment and protection from microbial pathogens, fungi, pests and predation [8]. The main constituents of essential oil of rose-scented geranium are acyclic monoterpenoids and acetate esters of monoterpenols [5]. The most abundant monoterpenoids are citronellol, geraniol, rose-oxide, linalool, and citronellyl formate [9]. The antioxidant, antibacterial, antifungal, antiviral, antiseptic, antidiabetic, antihemorrhoids and antitumor activities of the essential oils and their constituents have been widely studied [1, 10]. The distillate and absolute extracts (essential oil) from the foliage of the herb have a pleasant rose-like fragrance, and therefore are used as a substitute of expensive rose oil [11]. Further, Geraniaceae plants have been reported to synthesize and accumulate tartaric acid in leaves, possibly by ascorbate metabolism [12, 13]. Natural tartaric acid is a food additive serving as antioxidant, leavening agent, and flavor enhancer. Our group has developed a process for the production of scented natural tartaric acid from rose-scented geranium biomass as well as from residual water after hydro-distillation of the herb [13]. Thus, rose-scented geranium is a cash crop of high significance in pharmaceutical, food, phytoremediation, sanitary, cosmetic and perfume industries [14, 15].

There have been fewer molecular and biochemical studies on rose-scented geranium due to limited gene sequence information, as only 9 and 4 sequences were encountered on search of public domain nucleotide and protein databases, respectively, in NCBI GenBank dated December 21, 2016 (http://www.ncbi.nlm.nih.gov/Taxonomy/Browser/wwwtax.cgi?id=73200). Moreover, biochemical studies on the plant have been lacking as the plant was recognized as a hyper-acidic one [15]. Sangwan et al. [16] provided a process for isolation of proteins and catalytically active enzymes from rose-scented geranium. Next-generation sequencing (NGS) technologies have accelerated transcriptome investigations in several plant species, exploring qualitative and quantitative insights of global gene regulation [17]. In SRA database, raw sequencing reads are available for a total of 13 *Pelargonium* species: *P. tetragonum*, *P. fulgidum*, *P. transvaalense, P. incrassatum*, *P.austral*, *P. cotyledonis*, *P. nanum*, *P. citronellum*, *P. dichondrifolium*, *P. myrrhifolium*, *P. echinatum*, *P. exstipulatum*, and *Pelargonium x hortorum*. However, to date, transriptome information is not available for rose-scented species (https://www.ncbi.nlm.nih.gov/sra/?term=pelargonium). NGS has special significance in plants that produce low volume-high value specialized metabolites to advance their case for production through biotechnological approaches. Rose-scented geranium occupies a top-tier position in this list due to the metabolic characteristics of producing biomolecules of olfactory significance i.e. setero-isomers of monoterpenols and rose-oxide, one of the most attractive molecules of the aroma world. Terpenes are derived biosynthetically through terpenoids/isoprenoids pathway, wherein a five carbon phosphorylated isoprene moiety, isopentenyl pyrophosphate (IPP) and/or dimethyl allyl pyrophosphate (DMAPP), is the key building blocks of the diversified terpenoids. Recently, three genes from rose-scented geranium, *hydroxymethylglutaryl-CoA reductase* (*HMGR*), *1-deoxy-D-xylulose-5-phosphate synthase* (*DXS*), and *1-deoxy- D -xylulose 5-phosphate reductoisomerase* (*DXR*), which are related to isoprenoid biosynthesis, have been characterized in homologous as well as heterologous plant systems [18]. However, a massive pyrosequencing of transcriptome from rose-scented geranium is needed to get information of the putative genes and their transcriptional behavior in the metabolic pathways.

In this study, a comprehensive *de novo* transcriptome analysis of foliage of rose-scented geranium has been carried out. The transcriptional data provides a useful resource for functional genomic and molecular marker studies, and furthers our understanding of the biology of rose-scented geranium in general, and terpene and tartaric acid biosynthesis in particular.

## Methods

### Plant material

**Fig. 1:**
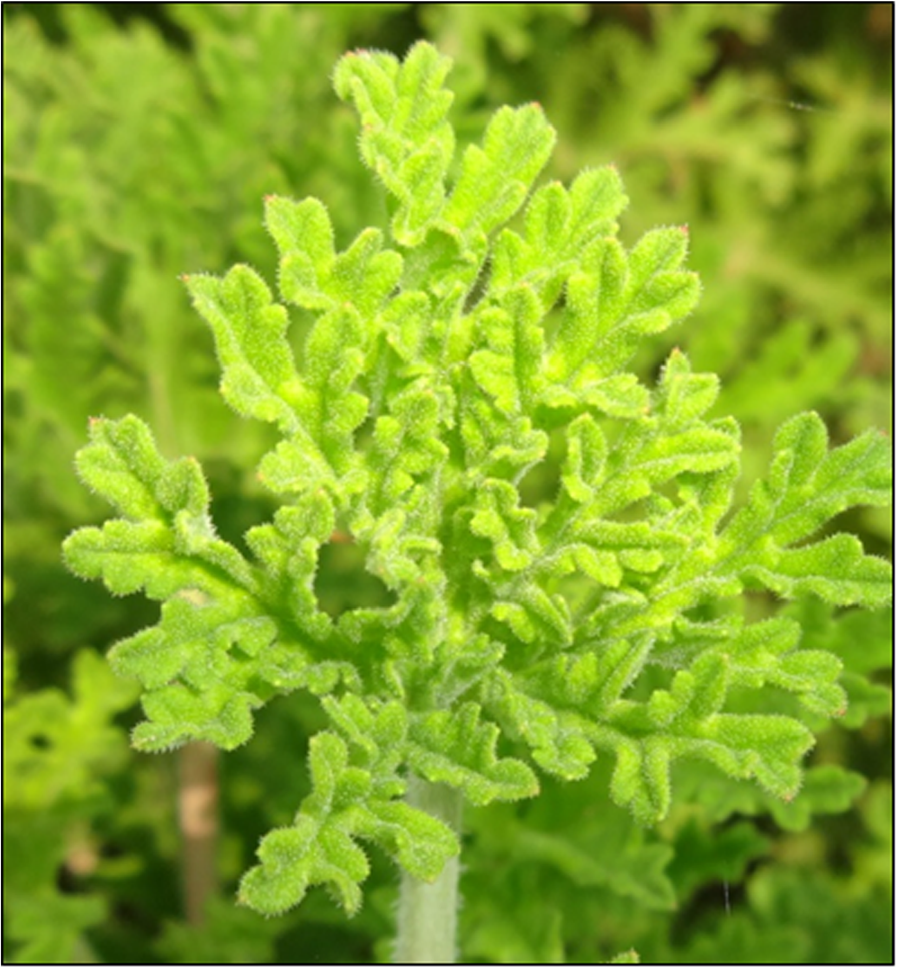
Field grown rose-scented geranium

Bourbon type rose-scented geranium (*Pelargonium* sp., family Geraniaceae) was used in this study. The Indian cultivars of rose-scented geranium are believed to be hybrids originating from *P. graveolens*, *P. radens* and *P. capitatum* [19]*.* Phylogenetic analysis, using the sequence of a plastid marker gene *trnL-F* in 57 *Pelargonium* species, placed rose-scented geranium cv. Bourbon close to *P. graveolens* (Additional file 1: Figure S1), which is in agreement with the morphological resemblance of Bourbon cultivar to this species [20]*.* Young leaves were collected from 2 to 3 month-old rose-scented geranium cv. Bourbon plants grown on the experimental field of Center of Innovative and Applied Bioprocessing (CIAB), Mohali, India (310 m above sea level; 30° 47′ N 76° 41′E) (Fig. 1). The samples were surface sterilized by using absolute ethanol and were immediately frozen in liquid nitrogen after harvest, and stored at −80 °C until use.

### RNA extraction and transcriptome sequencing

Total RNA was extracted from the leaf samples by a modified CTAB method, removing PVP from the extraction buffer and including a simple polyphenol and polysaccharide precipitation step to remove contaminating polyphenols and polysaccharides, as described by Asif et al. [21]. The quality and concentration of total RNA were determined by using Bioanalyzer (Model 2100, Agilent Technologies, USA). Total RNA, with an integrity number (RIN) of more than 8.0, from three biological replicates were pooled in equal amount and subjected to sequencing on the Illumina HiSeq 2500 platform (Illumina, USA), following standard protocols (http://www.illumina.com/). The transcriptome sequencing generated paired-end reads of 100 nt length.

### *De novo* assembly and expression analysis

The raw Illumina reads were processed for adaptor trimming and discard of low-quality reads by using NGS QC Toolkit (v2.3.3, NIPGR, India). High quality reads (Phred score >20) were assembled (*de novo*) into contigs using Trinity assembler (v2.0.6) at default parameters, which have been shown to provide relatively better assembly of Illumina data with deep transcriptome coverage in the absence of a reference genome [22]. The assembled contigs, longer than 200 bp, were clustered by using CD-HIT tool (v4.6.1) to obtain non-redundant contigs [23]. Transcript assembly was validated by mapping the high quality reads to the assembled contigs by using BOWTIE2 (1.0.0) software at default parameters, as explained in Bankar et al. [24]. The assembly-validated file was processed by using Bedtools and Samtools for read count estimation (quantitation), as explained in Bankar et al. [24]. RSEM software was used for normalization of mapped reads, and TPM (tags per million) and FPKM (fragments per kilobase per million) were obtained. Log2 transformed FPKM values were considered as absolute expression of the transcripts.

### Functional annotation

Putative function was assigned to each transcript by using BLASTx homology search against non-redundant (NR) protein database, at the criteria of e-value <0.001 and query coverage above 50%. NR BLAST hits were used to derive associated Gene Ontology (GO) terms from UniProt database. Transcription factors and hormone related transcripts were identified by doing BLASTx against all plant transcription factors database (Plant-TFDB 3.0; http://planttfdb.cbi.pku.edu.cn/), and *Arabidopsis thaliana* hormone database (http://molbio.mgh.harvard.edu/sheenweb/Ara_pathways.html), at e-value 1e^−5^ and query coverage 50%. In addition, BLAST hits (e-value cut off 1e^−5^ and query coverage at least 50%) against *A. thaliana* protein database (ftp://ftp.psb.ugent.be/pub/plaza/plaza_public_dicots_03//Fasta/proteome.ath.tfa.gz) were used for MapMan (v3.6.0RC1) functional categorization of transcripts.

### SSRs identification

Assembled contigs were searched for detection of SSRs by using MISA (MIcroSAtellite) tool (http://pgrc.ipk-gatersleben.de/misa/) at default parameters. A minimum of five repetitions was considered as search criteria in MISA script for identification of mono- to hexa-nucleotide motifs. Both perfect (contain a single repeat motif) and compound repeats (composed of two or more motifs) were identified.

### Experimental validation of transcriptome assembly

A total of four putative genes were randomly selected for wet lab assembly validation namely; 1-deoxy-D-xylulose 5-phosphate reductoisomerase, zeaxanthin epoxidase, WRKY-4 and GDP mannose 3′, 5′ epimerase by using the primers designed on the basis of the sequence of the assembled transcript. Standard PCR reactions were conducted using cDNA prepared from young leaf and Dream-taq PCR master mix (Thermo Scientific, USA). The details of the primers used for amplifying respective fragments are mentioned in Additional file 2.

### Validation of gene expression by semi quantitative and quantitative real time PCR analyses

The quantitation of randomly selected transcripts from RNA-seq data was validated by semi quantitative and real time PCR assays. The expression analysis was performed for 12 genes belonging to terpene biosynthesis pathway, tartaric acid pathway, transcription factor and hormone biosynthesis pathway *viz* 1-deoxy-D-xylulose 5-phosphate reductoisomerase, geranyl diphosphate synthase, farnesyl pyrophosphate synthase, linalool synthase, hexokinase, GDP-mannose-3′,5′-epimerase, L- idonate 5-dehydrogenase, polygalacturonase, WRKY-4, MYB, zeaxanthin epoxidase and cytochrome P_450_ for expression analysis. Real-time PCR was carried out in three independent biological replicates and three technical replicates by using SYBR Green master mix (Applied Biosystems, USA). Actin gene was used as internal control to normalize the expression. Semi quantitative PCR reactions were conducted using Dream-taq PCR master mix (Thermo Scientific, USA). The details of the primers used for semi quantitative and real-time PCR are mentioned in Additional file 2.

## Results and discussion

### *De novo* assembly and functional annotation

**Fig. 2:**
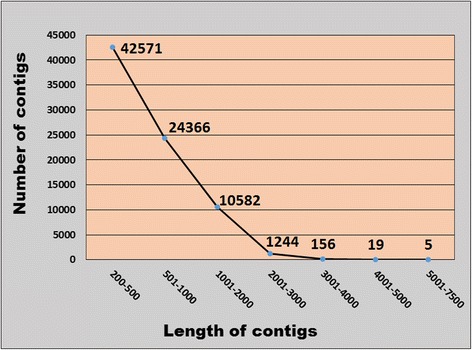
Distribution of rose-scented geranium contigs according to their size

**Fig. 3:**
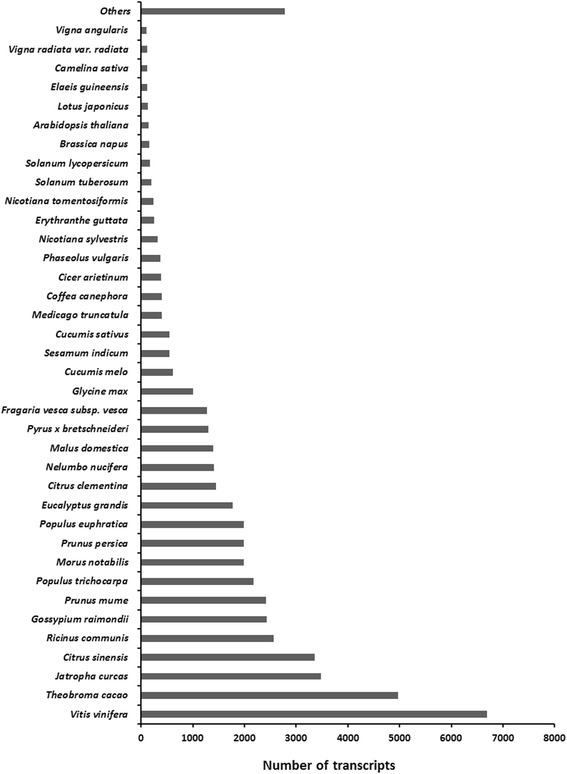
Distribution of the top hits for unique proteins in NR database

*De novo* RNA-seq approach facilitates analysis of transcriptome for an organism without sequenced genome such as rose-scented geranium [21]. Transcriptome sequencing of rose-scented geranium foliage on Illumina platform generated a total of 16.05 million raw reads. The filtered reads were deposited in NCBI Short Read Achieve (SRA) database under accession number SRP078041. A total of 15.44 million high quality reads were *de novo* assembled into 78,943 nonredundant contigs (>200 bp length), with an average length of 623 bp and N50 length of 752 bp (Table 1). The total size of the assembled transcriptome was amounted as 49.23 Mb, with average GC content of 44.97%. Majority of the contigs (53.92%) had 200 to 500 bp lengths. The lengths of 30.86% contigs (24,366) were ranged from 501 to 1000 bp, followed by 14.98% contigs (11,826) of 1001–3000 bp. Only 24 transcripts were detected in the range of 4001–7500 bp (Fig. 2). All the transcripts of the rose-scented geranium were searched (BLASTx) against known proteins in NR database, annotating a total of 51,802 contigs. A total of 611 plant species contributed the annotated contigs in the top-scoring BLASTx hits against NR protein database (Additional file 3). Out of these, top five species that contributed the greatest number of annotated contigs were *Vitis vinifera*, *Theobroma cacao, Jatropha curcas*, *Citrus sinensis*, and *Ricinus communis* (Fig. 3). The results provided transcript sequence information, their expression and putative function of the genes expressed in the leaves of rose-scented geranium (Additional file 3)*.* The transcriptome data is a useful resource for identifying genes with putative roles in various biochemical activities and pathways in the volatile oil plant.

**Table 1:** Summary of the sequencing-reads, assembly and functional annotation of rose-scented geranium transcriptome

Parameters	Counts
Total reads	16,051,328
High quality (phred score >20) reads	15,444,409
Total number of nonredundant contigs (≥200 bp)	78,943
Average contigs length (bp)	623
N50 (bp)	752
(G + C)%	44.96%
Annotated contigs	51,802

### Functional categorization

The contigs having sequence homology with uniprot annotations were subjected to GO assignments under biological processes, cellular component and molecular function categories. A total of 25,776 transcripts were assigned to at least one GO term (Additional file 4). In the category of biological processes, transcripts related to transcription regulation, translation, carbohydrate metabolic process, transmembrane and intracellular protein transports were predominant. In molecular functions, genes involved in ATP binding, DNA binding, zinc ion binding, nucleic acid binding and structural constituent of ribosome were abundantly expressed. In cellular components, genes related to integral component of membrane, nucleus, intracellular, cytoplasm and ribosome were the most abundant classes (Additional file 1: Figure S2).

A total of 54,104 rose-scented geranium contigs could be mapped to 12,381 non-redundant *A. thaliana* protein sequences (Additional file 5). The orthologous *A. thaliana* gene ids were used to perform MapMan analysis. MapMan results visualized significant representation of genes associated with secondary metabolic biosynthesis pathways as terpenes, flavonoids, and phenylpropanoids (Additional file 1: Figures S3 and S4). The secondary metabolites participate in active defense mechanism of plants providing protection from a wide range of stresses [25]. Accordingly, MapMan analysis revealed putative genes quoted as involved in biotic and abiotic stress responses (Additional file 1: Figure S5).

### Terpene biosynthesis

Rose-scented geranium produces essential oil, containing fragrant as well as other specialized metabolites with antioxidant, antimicrobial, and human health-promoting effects, in specialized tissues of leaves known as glandular trichomes. Terpenes are the largest and the most diverse class of natural products, and constitute a major component of essential oil in rose-scented geranium. They are produced as a homologous series of molecules as polymers of isoprene, the C_5_ precursor molecules being IPP and/or DMAPP that are generated via the process of isoprenogenesis [11, 26]. In plants, isoprenogenesis occurs through two discrete biosynthetic pathways: the mevalonic acid (MVA) pathway in cytosol and the 2-C-methyl-D-erythritol 4-phosphate/1-deoxy-D-xylulose 5-phosphate (MEP/DOXP) pathway in plastids. Their relative contribution for isoprenes, to be used in terpenoid biosynthesis, depends on many factors such as specific sub-classes of terpenoids, specific terpenoidal molecules, quantitative level of production and environmental conditions. Generally, the MEP/DOXP pathway generates monoterpenes and diterpenes, whereas the MVA pathway is largely responsible to produce sesquiterpenes and triterpenes [27]. However, there are exceptions to this generalization and exchange of precursors as well between the two pathways [28], for example, the MEP/DOXP pathway synthesizes sesquiterpenes along with monoterpenes in *Antirrhinum majus* [29].

**Fig. 4:**
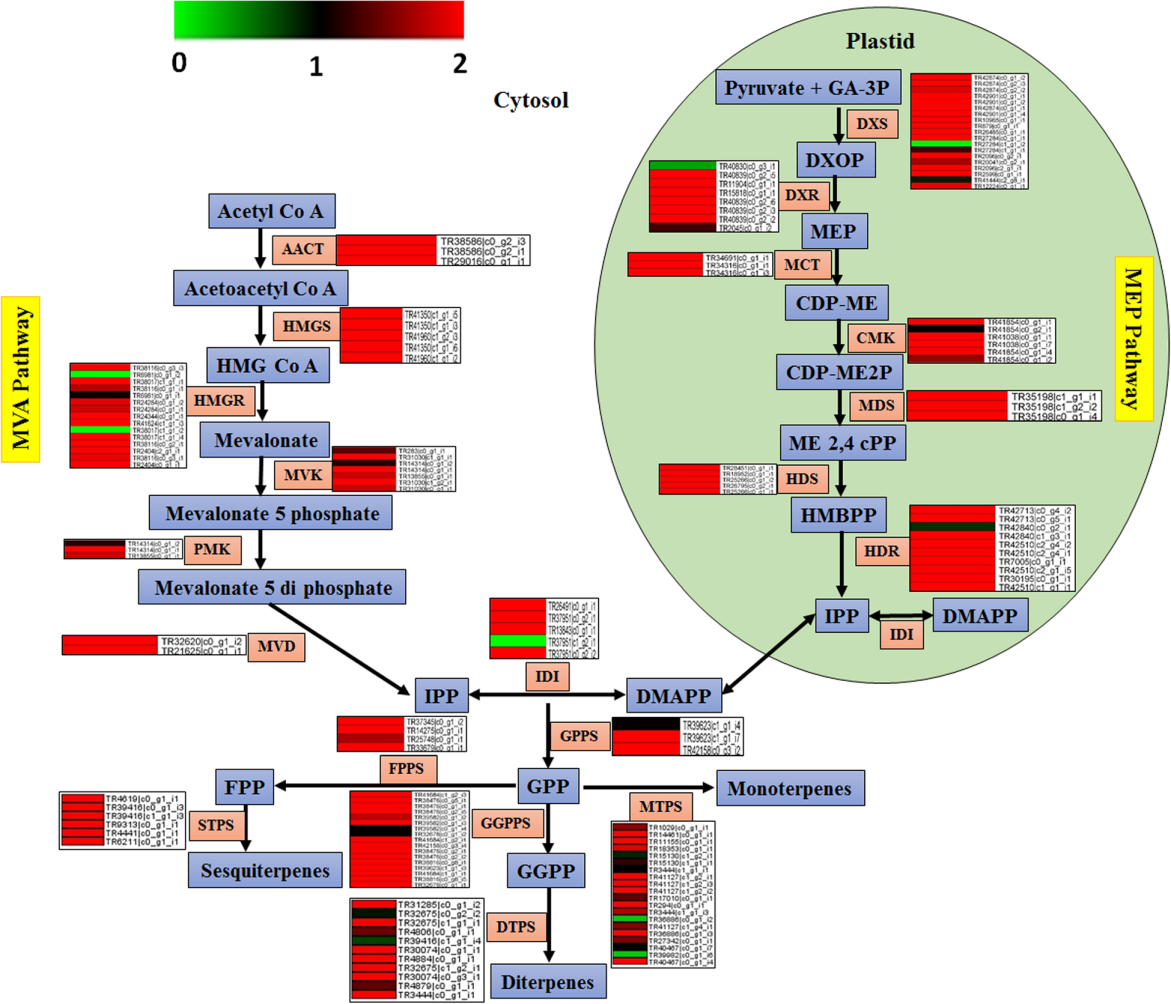
Schematic representation of terpene biosynthetic pathway, and heatmaps displaying the expression (log2 FPKM) of enzymes involved in the different reaction steps. The details of the transcripts are given in Additional file 6. AACT, acetoacetyl-CoA thiolase/acetyl-CoA acetyltransferase; HMGS, hydroxymethylglutaryl- CoA synthase; HMGR, hydroxymethylglutaryl-CoA reductase; MVK, mevalonate kinase; PMK, phosphomevalonate kinase; MVD, mevalonate diphosphate decarboxylase; DXS, 1-deoxy- D -xylulose 5-phosphate synthase; DXR, 1-deoxy- D -xylulose 5-phosphate reductoisomerase; MCT, 2-C-methyl-D-erythritol 4-phosphate cytidylyltransferase; CMK, 4-(cytidine 5′-diphospho)-2-C-methyl-D-erythritol kinase; MDS, 2-C-methyl- D -erythritol 2,4-cyclodiphosphate synthase; HDS, (E)-4-hydroxy-3-methylbut-2-enyl diphosphate synthase; HDR, (E)-4-hydroxy-3-methylbut-2-enyl diphosphatereductase; GPPS, geranyl diphosphate synthase; IDI, isopentenyl-diphosphate delta isomerase; FPPS, farnesyl pyrophosphate synthase; GGPPS, geranylgeranyl diphosphate synthase; MTPS, mono-terpene synthase; STPS, sesqui-terpene synthase; DTPS, di-terpene synthase; HMG, CoA, hydroxymethylglutaryl-CoA; IPP, isopentenyl pyrophosphate; DMAPP, dimethylallyl pyrophosphate; GA-3P, glyceraldehyde 3-phosphate; DXOP, 1-deoxy-D-xylulose-5-phosphate; MEP, 2-C-methyl-d-erythritol-phosphate; CDP-ME, 4-(cytidine5′ -diphospho)-2-C-methyl-d-erythritol; CDP-ME2P, 2-phospho 4- (cytidine 5′-diphospho)2-c-methyl-d-erythritol; ME 2,4 cPP, C-methyl-D-erythritol 2,4-cyclodiphosphate; HMBPP, 1-hydroxy-2-methyl-2-butenyl 4-diphosphate; GPP, geranyl pyrophosphate; FPP, farnesyl pyrophosphate; GGPP, geranylgeranyl pyrophosphate; MVA, mevalonic acid

In MVA pathway, IPP is biosynthesized by sequential actions of acetoacetyl-CoA thiolase/acetyl-CoA acetyltransferase (AACT), hydroxymethylglutaryl- CoA synthase (HMGS), hydroxymethylglutaryl-CoA reductase (HMGR), mevalonate kinase (MVK), phosphomevalonate kinase (PMK), and mevalonate diphosphate decarboxylase (MVD) (Fig. 4). AACT condenses two molecules of acetyl CoA to biosynthesize acetoacetyl CoA, and then HMGS combines acetyl CoA with acetoacetyl CoA generating hydroxymethylglutaryl CoA (HMG-CoA) [30]. The transcriptome analysis identified three unique putative genes for AACT (e-value: 1e^−48^ to 0) and four for HMGS (e-value: 1e^−30^ to 0) in rose-scented geranium. A total of thirteen unique putative transcripts represented NADPH-dependent enzyme- HMGR (e-value: 3e^−21^ to 0) in rose-scented geranium, which catalyzes the biosynthesis of mevalonate from HMG-CoA [17, 31]. The sequence analysis of putative AACT, HMGS and HMGR genes suggested that they contain full-length open reading frames (ORFs). Mevalonate is transformed into mevalonate 5- di phosphate by two phosphorylation reactions catalyzed by MVK and PMK. Thereafter, MVD converts mevalonate 5- di phosphate into the key isoprene unit, IPP. The transcriptome examination revealed homologies with four MVK (e-value: 2e^−41^ to 6e^−126^), one PMK (e-value: 2e^−41^ to 3e^−52^), and two MVD (e-value: 2e^−47^ to 9e^−51^) putative unique genes. IPP is enzymatically isomerized into DMAPP by isopentenyl diphosphate isomerase (IDI), and thus providing two types of phosphorylated isoprenes (IPP and DMAPP) for isoprenoid biosynthesis. The transcriptome analysis identified five representative contigs for IDI (e-value: 1e^−56^ to 5e^−129^). Sequence analysis suggested presence of complete ORFs in the putative IDI gene.

In DOXP pathway, biosynthesis of IPP or DMAPP involves seven enzymatic steps (Fig. 4). The condensation of pyruvate and D -glyceraldehyde 3-phosphate (GAP) is catalyzed by 1-deoxy- D -xylulose 5-phosphate synthase (DXS), producing 1-deoxy- D -xylulose-5-phosphate (DOXP) that is transformed into 2-C-methyl-D-erythritol 4-phosphate (MEP) by 1-deoxy- D -xylulose 5-phosphate reductoisomerase (DXR) or MEP synthase [17]. A total of 9 and 8 unique putative genes were identified related to DXS (e-value: 2e^−24^ to 0) and DXR (e-value: 3e^−29^ to 0), respectively. Computational analysis predicted full-length sequences of the candidate protein-coding DXS and DXR genes. The enzyme 2-C-methyl-D-erythritol 4-phosphate cytidylyltransferase (MCT) catalyzes conversion of MEP into 4-(cytidine 5′ -diphospho)-2-C-methyl- D-erythritol (CDP-ME), which is then transformed into 2-phospho 4- (cytidine 5′ -diphospho) 2-C-methyl-d-erythritol (CDP-ME2P) by 4-(cytidine 5′-diphospho)-2-C-methyl-D-erythritol kinase (CMK). The enzymatic actions of 2-C-methyl- D -erythritol 2,4-cyclodiphosphate synthase (MDS) and (E)-4-hydroxy-3-methylbut-2-enyl diphosphate synthase (HDS) causes sequential conversion of CDP-ME2P into C-methyl-D-erythritol 2,4-cyclodiphosphate (ME 2,4 cPP), and then 1-hydroxy-2-methyl-2-butenyl 4-diphosphate (HMBPP). Finally, biosynthesis of IPP happens from HMBPP by (E)-4-hydroxy-3-methylbut-2-enyl diphosphate reductase (HDR) [30]. The transcriptome investigation identified three unique putative contigs for CMK (e-value: 2e^−17^ to 5e^−146^), two for MDS (e-value: 1e^−29^ to 3e^−91^), three for HDS (e-value: 1e^−41^ to 6e^−77^), and five for HDR (e-value: 7e^−27^ to 4e^−110^). The putative CMK and MDS genes showed full-length ORFs in sequence analysis.

The C5 units, IPP or DMAPP, may be linked together by head to tail condensation reaction resulting terpenes of different classes e.g. mono, sesqui, di and triterpenes. The first condensation step of IPP and DMPP is catalyzed by geranyl diphosphate synthase (GPPS), synthesizing geranyl pyrophosphate (GPP). GPP is substrate for monoterpene biosynthesis by enzymatic actions of monoterpene synthases (MTPS), such as geraniol synthase and linalool synthase. Catalysis of sequential coupling of IPP units to GPP results farnesyl pyrophosphate (FPP) and geranylgeranyl diphosphate (GGPP) by farnesyl pyrophosphate synthase (FPPS) and geranylgeranyl diphosphate synthase (GGPPS) enzymes, respectively. FPP and GGPP are substrates for sesquiterpene and diterpene biosynthesis, catalyzed by sesquiterpene synthases (STPS) and diterpene synthases (DTPS) [32, 33]. The transcriptional profiling identified two representative unique transcripts for GPPS (e-value: 1e^−54^ to 2e^−146^), three for FPPS (e-value: 2e^−56^ to 8e^−155^), ten for GGPPS (e-value: 5e^−21^ to 2e^−164^), thirteen for MTPS (e-value: 1e^−32^ to 0), five for STPS (e-value: 9e^−20^ to 6e^−166^), and ten unique contigs for DTPS (e-value: 3e^−14^ to 1e^−106^). Full-length sequences were obtained in case of the candidate genes for GGPPS, MTPS (ocimene synthase) and STPS (germacrene D synthase).

The essential oil of rose scented geranium contains several mono-, di and sesquiterpenes. The main components which determine its aroma are citronellol, geraniol, linalool and their esters [34]. In addition, significant quantities of isomenthone, menthone, nerol, *cis*-and *trans*-rose oxides, α-terpineol, α -pinene, myrcene, and β-phyllandrene contributes to its aroma [26]. In agreement with the aroma profile of this plant, significant level of expression was observed for the putative genes encoding geraniol synthase, linalool synthase, myrcene synthase, β-ocimene synthase, limonene synthase, germacrene synthase, nerolidol synthase, cadinene synthase, copalyl diphosphate synthase, kaurene synthase, and BAHD acyltransferase.

In the annotated rose-scented geranium leaf transcriptome, a total of 158 contigs were mapped on 103 unique proteins involved in terpene biosynthesis, with significantly low e-value (Fig. 4; Additional file 6). The putative protein-coding genes exhibited presence of conserved ORFs, and many of them were likely to contain complete ORFs, suggesting identification of relevant transcripts involved in the terpene biosynthetic pathways. The putative genes involved in downstream steps of the MEP pathway exhibited relatively higher expression as compared to the MVA pathway (Additional file 6), which is in agreement with abundance of monoterpene hydrocarbons in essential oil of geranium plants [5, 27]. The sequence information and transcriptional pattern of the putative genes would be useful in understanding molecular mechanism and engineering of terpene biosynthesis in rose-scented geranium*.*

### Tartaric acid biosynthesis pathway

**Fig. 5:**
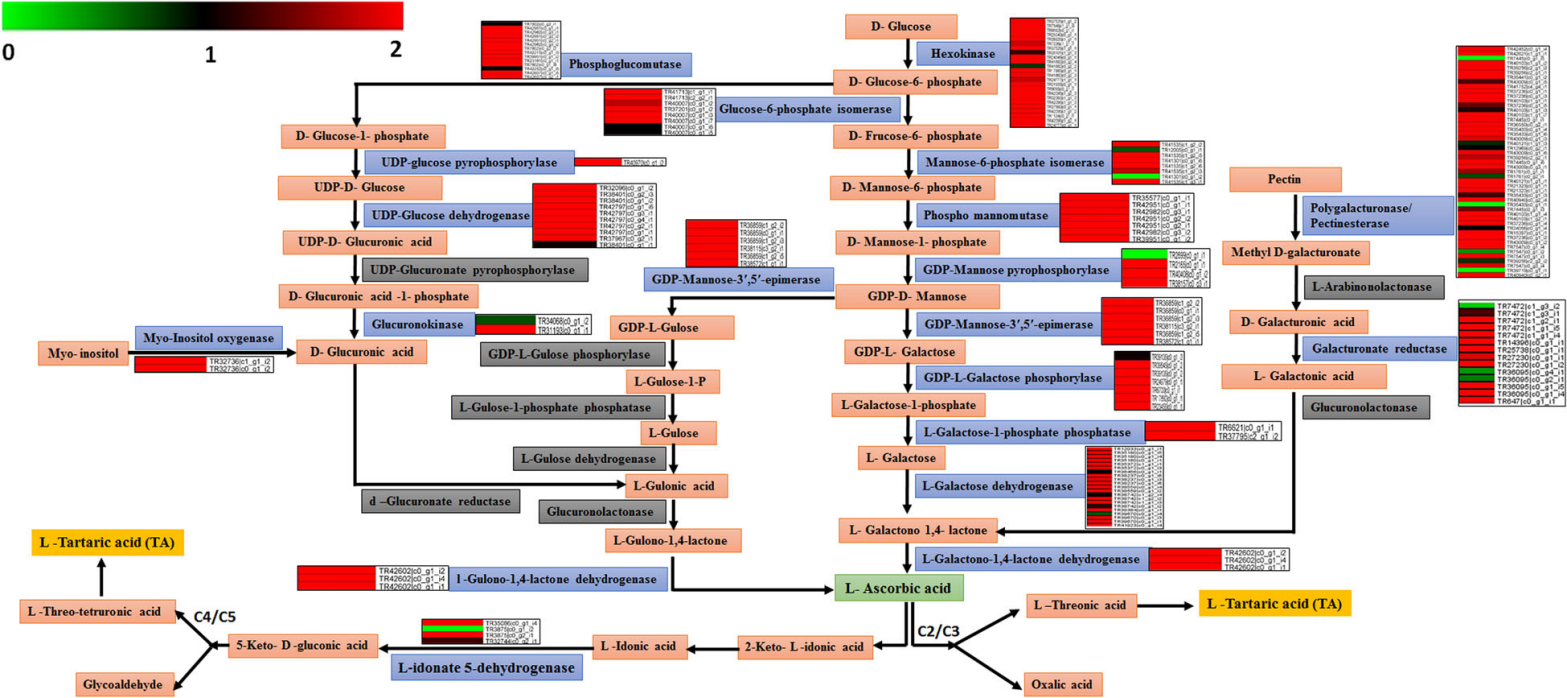
Schematic representations of ascorbic acid and tartaric acid biosynthesis, and heatmaps displaying the expressed transcripts (log2 FPKM) related to enzymes involved in the different reaction steps. Transcripts were not detected for the enzymes represented in gray color. The details of the transcripts are given in Additional file 7

The plant-derived metabolite, tartaric acid, is of high human value as a vital antioxidant and flavorant in food products. Recently, our group established a process for production of scented natural tartaric acid from rose-scented geranium biomass *per se* or from residual water after hydro-distillation of the geranium foliage [13]. Ascorbic acid (vitamin C), the most abundant soluble antioxidant in cells of higher plants, is a putative biosynthetic precursor in the formation of tartaric acid. Tartaric acid biosynthesis is the result of catabolism of the six-carbon ascorbic acid. The hydrolysis of ascorbic acid may follow cleavage between the carbon atoms 2 and 3 or 4 and 5, with still unresolved plant-species specific preference of the alternative cleavage pathways [35]. The 2–3 cleavage in ascorbic acid results oxalic acid and threonic acid, further oxidizes into tartaric acid [36]. Alternatively, ascorbic acid is converted to idonic acid, and the latter into an intermediate compound 5-keto D-gluconic acid by the action of an enzyme called idonate dehydrogenase. The intermediate compound is then cleaved between carbon atoms 4 and 5 resulting tartaric acid [12]. Though, intermediates of tartarate biosynthesis from ascorbic acid have been characterized chemically, enzymes catalyzing all the reactions are yet to be identified. Geraniaceae family plants have been suggested to follow C2-C3 cleavage in ascorbic acid during tartarate biosynthesis [12, 35, 36]. However, no enzymatic or genomic information about the metabolic steps is known. The transcriptome analysis of rose-scented geranium notified substantial level of expression for idonate dehydrogenase (IDH) (Fig. 5). The sequence analysis of IDH gene revealed 80% protein sequence identity with that of *Vitis venifera* (XP_010662490) at 99% query coverage and zero e-value. As IDH is involved in C4-C5 cleavage of ascorbate [35], the findings indicate the possibility of operation of both the C2/C3 and C4/C5 pathways of ascorbic acid hydrolysis for tartarate biosynthesis in rose-scented geranium*.*

Smirnoff-Wheeler pathway is the principal route for biogenesis of the precursor multifunctional metabolite ascorbic acid in higher plants [37, 38]*.* Smirnoff-Wheeler pathway is based on photosynthesis-based carbon flux and catalyzed by a series of enzymes, such as GDP-D-mannose 3′, 5′ epimerase (ME), GDP-L-galactose phosphorylase (GP), L-galactose-1-phosphate phosphatase (GPP), L-galactose dehydrogenase (GD), and L-galactono-1,4-lactone dehydrogenase (GLDH) [39]. The transcriptome investigation identified six unique putative genes representing ME (e-value: 8e^−47^ to 0), four for GP (e-value: 1e^−24^ to 2e^−117^), one for GPP (e-value: 8e^−46^ to 9e^−64^), sixteen for GD (e-value: 1e^−28^ to 0), and one putative gene for GLDH (e-value: 2e^−122^ to 0). Full-length transcripts with relevant putative ORFs were obtained for the aforementioned key enzymes involved in ascorbate biosynthesis. Transcripts were also identified for two other ascorbic acid biosynthetic routes arising from *myo*-inositol and pectin (Fig. 5), as reported in few plants [35]. A total of 189 contigs could be mapped on 130 unique genes belonging to ascorbic acid and tartaric acid biosynthesis (Additional file 7).

### Anacardic acid biosynthesis pathway

**Fig. 6:**
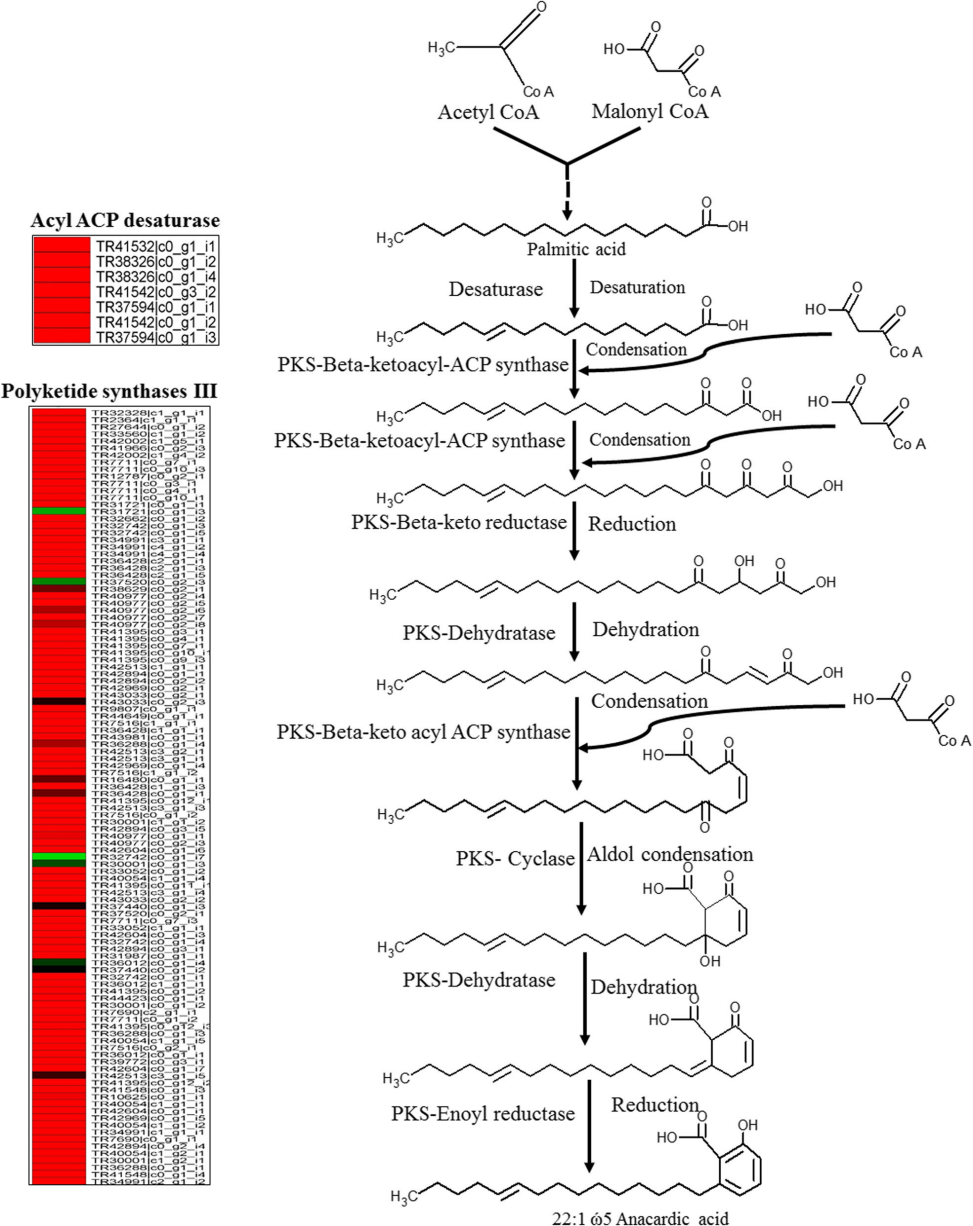
Schematic representation of anacardic acid biosynthesis, and heatmaps displaying the expressed transcripts (log2 FPKM) related to enzymes involved in the different reaction steps. The details of the transcripts are given in Additional file 8

Anacardic acid (2-hydroxy-6-alkylbenzoic acid) is a dietary and medicinal phytochemical structurally similar to salicylic acid. It has been reported to be produced in glandular trichomes of Geraniaceae plants, conferring pest resistance [40–42]. Pest resistant and susceptible genotypes exhibit predominance of unsaturated (22:1 ώ^5^ and 24:1 ώ^5^) and saturated (22:0 and 24:0) anacardic acid, respectively [40, 43]. The biosynthesis of anacardic acid could happen through polyketide mechanism using fatty acids as precursor molecules [41, 44]. Carbon elongation in anacardic acid is achieved by utilizing acetate units derived from malonyl-CoA. Acyl-[acyl-carrier-protein] desaturase and type III polyketide synthase (PKS), a muilti module protein, catalyzes condensation reaction steps of anacardic acid biosynthesis [41]. In transcriptome data, a total of 114 contigs were identified, with substantial level of expression, showing homology with thirteen type-III PKS (e-value: 3e^−19^ to 0) and six Acyl ACP desaturase (e-value: 1e^−53^ to 5e^−175^) putative genes (Fig. 6; Additional file 8). However, their involvement as candidate genes in anacardic acid biosynthesis need to be further validated.

### Putative genes for transcription factors and hormones

**Fig. 7:**
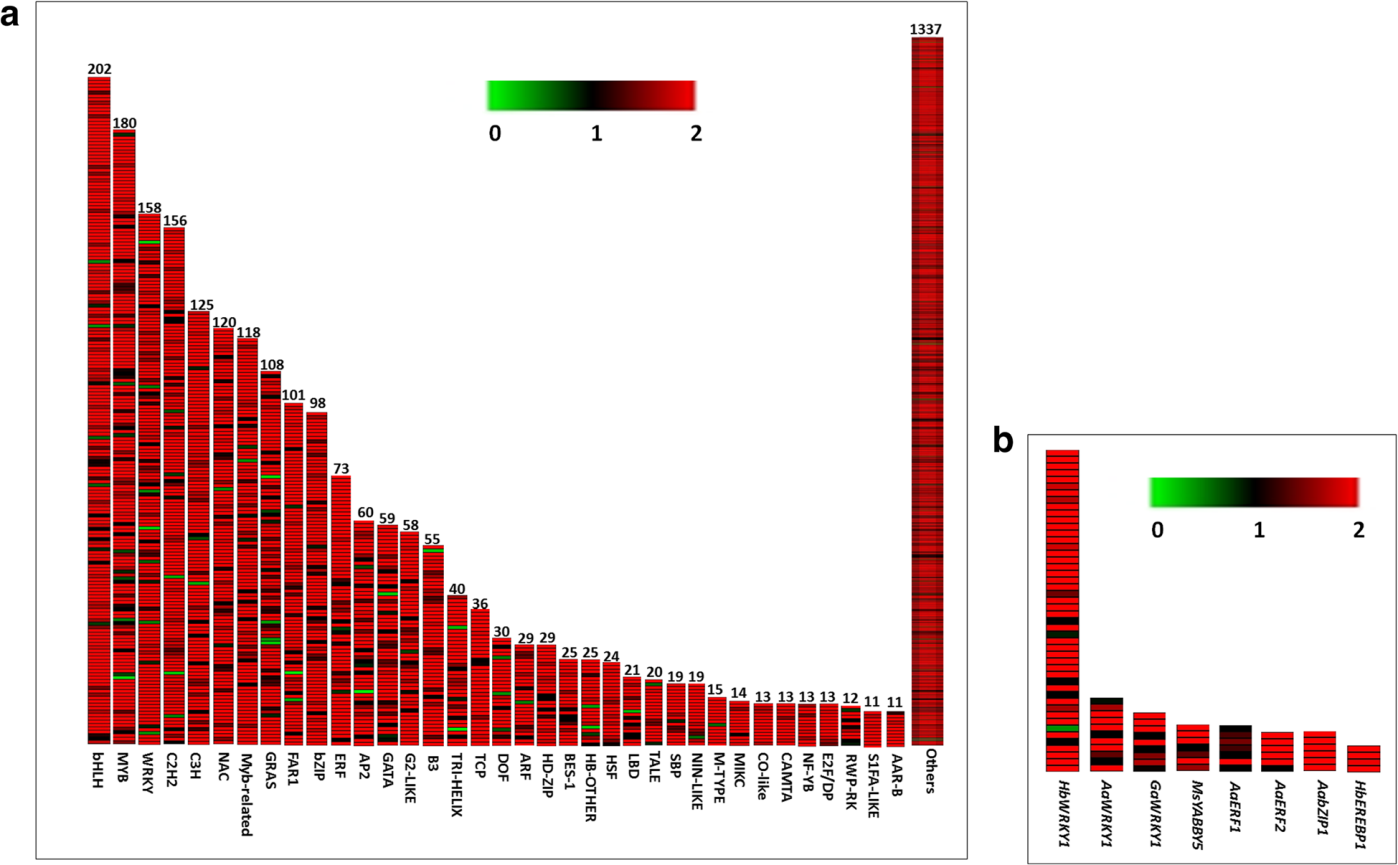
Putative orthologous TF genes (>10) belonging to different TF families (**a**), and putative TF genes regulating terpene biosynthesis (**b**). The details of the transcripts are given in Additional file 9

Transcription factors (TFs) modulate, qualitative and quantitative transcriptional behavior of genes at spatial and temporal level regulating various metabolic pathways. The rose-scented geranium contigs were annotated against all plant TFs. A total of 15,666 contigs were mapped on 3,440 all plant TF genes at an e-value cut-off of 1e^−5^ (Additional file 9). The putative genes for the TF families- bHLH, MYB, WRKY, C2H2, CH, NAC, MYB-related, GRAS, FAR1, and bZIP were significantly abundant in the transcriptome data (Fig. 7a). The TFs belonging to bHLH, MYB, AP2/ERF, and WRKY families execute key roles in regulation of biosynthesis of terpenes, which is the largest secondary metabolite family [45–49]. Some of the TF genes have been characterized for regulating biosynthesis of terpene secondary metabolites in different plants e.g. *AaWRKY1*, *AaERF1*, *AaERF2*, *AaORA1*, *AabZIP1*, *GaWRKY1*, *TaWRKY1*, *HbEREBP1*, *HbWRKY1*, *OsTGAP1*, and *MsYABBY5* [50]. Transcripts were identified for orthologous putative genes of these TFs (e-value: 3e^−8^ to 0) in the transcriptome data (Fig. 7b; Additional file 9). Some of the putative TFs exhibited presence of complete ORFs in the sequence e.g. *AaWRKY1, AabZIP1, GaWRKY1*, *HbWRKY1,* and *MsYABBY5*. These could be potential candidates for metabolic engineering and improvement of the production of secondary metabolites in rose-scented geranium*.*

**Fig. 8:**
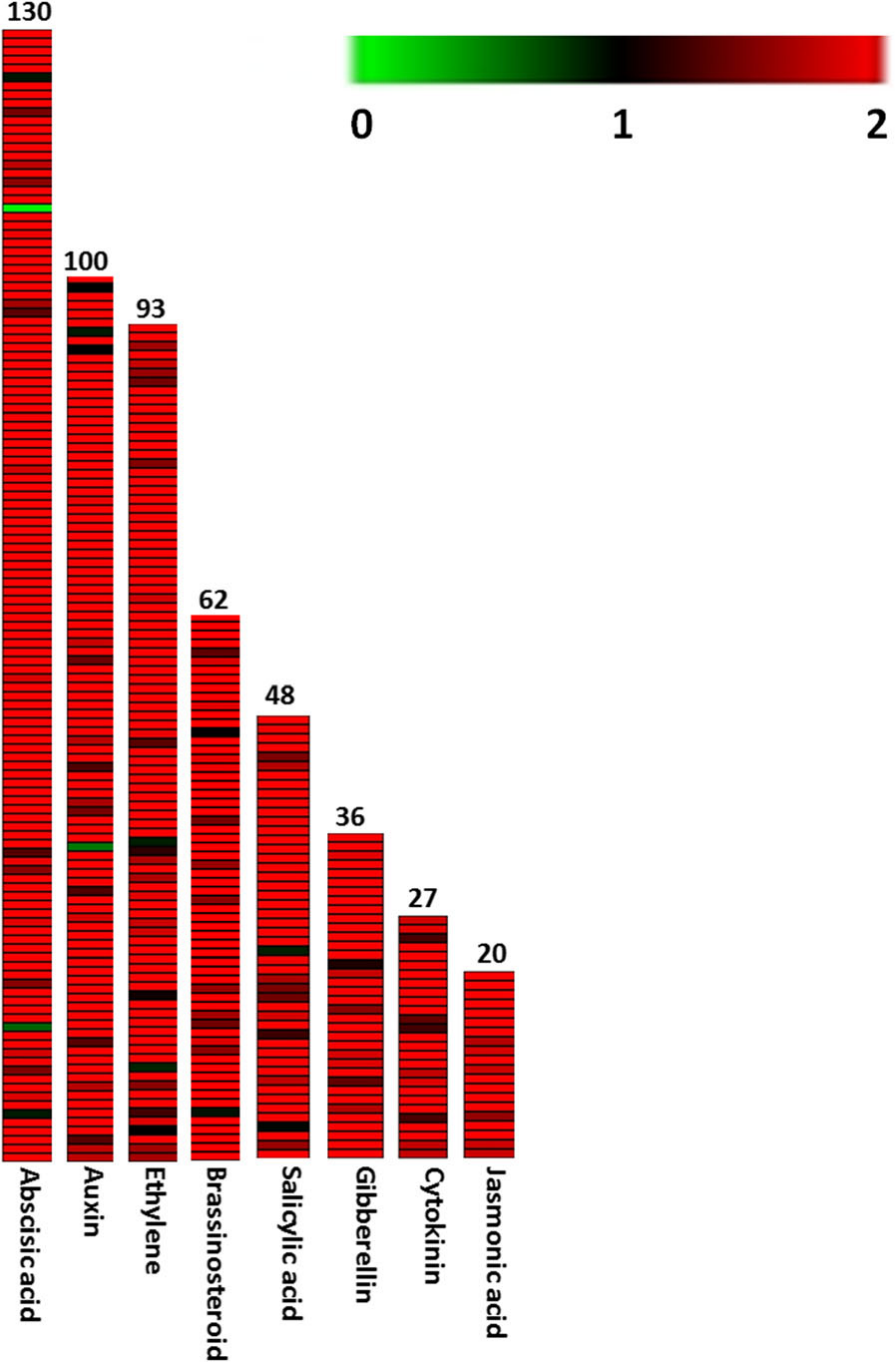
Putative orthologous genes related to different hormones. The details of the transcripts are given in Additional file 10

Signaling molecules known as phytohormones regulate the plant development and physiological processes, and responses to environment and endogenous factors. In the transcriptome data, a total of 9,453 rose-scented geranium transcripts were matched with 516 protein sequences (e-value >1e^−5^) of *A. thaliana* belonging to different hormones: abscisic acid, auxin, ethylene, brassinosteroid, salicylic acid, gibberellin, cytokinin, and jasmonic acid (Fig. 8; Additional file 10). When analyzed hormone related transcripts with ≥ 5 Log2 FPKM, orthologous genes for abscisic acid, ethylene, auxin and brassinosteroids were found most abundant, followed by salicylic acid, jasmonic acid and gibberellin (Additional file 10). Plant hormones such as jasmonic acid, salicylic acid, and abscisic acid have been noted as potential elicitors of secondary metabolite biosynthesis [48, 51].

The sequence and transcriptional pattern information of TFs and hormones would be useful in understanding secondary metabolism as well as engineering of biosynthesis of value-added compounds (e.g. terpene and tartaric acid) in rose-scented geranium.

### SSR detection

Simple sequence repeats (SSR) are locus-specific, co-dominant, abundant in genome, and multiallelic molecular markers with high rates of transferability across the species [52, 53]. Mining of SSRs was carried out to enable the development of molecular markers in rose-scented geranium*.* In total, 6040 SSR motifs (mono to hexa nucleotides) were identified in 5380 contigs (Additional file 11), which corresponds to 6.8% of the total unique transcripts. The result agrees with previous studies reporting approximately 3–7% of expressed sequences with putative SSR motifs [54]. Out of these analyzed transcripts, 571 contained more than one SSR, whereas, 316 were in compound form (Table 2). The highest frequency of SSR was of tri-nucleotide (50%), followed by di- (28.13%) and mono-nucleotide (18.95%), as represented in Table 2. These SSR motifs could be potential candidates for development of transcript based microsatellite marker, helpful in analyzing molecular mapping, marker assisted selection, and functional genetic variation in rose-scented geranium and related *Pelargonium* species.

**Table 2:** Statistics of SSRs discovered and various classes of SSR repeat motifs in rose-scented geranium transcriptome

Parameters	Counts	Motifs	Number of contigs
Total number of sequences examined	78943	Mono-nucleotides	1145
Total number of identified SSRs	6040	Di-nucleotides	1699
Number of SSR containing sequences	5380	Tri-nucleotides	3011
Number of sequences containing more than one SSR	571	Tetra-nucleotides	71
Number of SSRs present in compound formation	316	Penta-nucleotides	52
		Hexa-nucleotides	62

### Assembly validation

The *de novo* transcriptome assembly, done by Trinity assembler tools, was validated by using standard PCR. End-to-end primers were designed using sequences of four randomly selected putative genes of different size *viz* 1-deoxy-D-xylulose 5-phosphate reductoisomerase (689 bp), GDP mannose 3′, 5′ epimerase (799 bp), WRKY-4 (992 bp) and zeaxanthin epoxidase (369 bp). PCR assay, using first strand cDNA of rose scented geranium leaf as template, followed by agarose gel electrophoresis yielded amplicons of expected size of the respective transcripts (369 to 992 bp), validating transcriptome assembly (Additional file 1: Figure S6).

### Validation of putative gene expression via semi-quantitative and real-time PCR

**Fig. 9:**
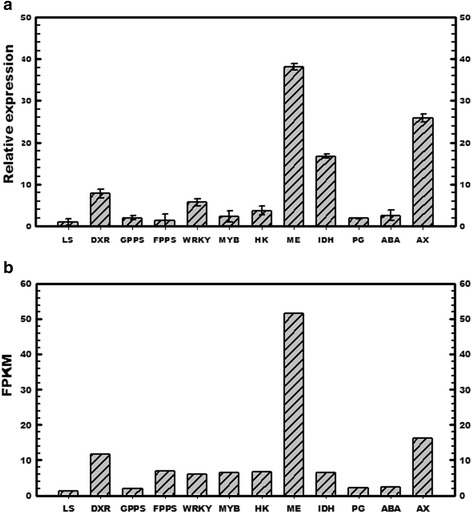
Quantitative real time PCR analysis (**a**) to validate FPKM expression values (**b**) of randomly selected contigs from rose-scented geranium leaf transcriptome. DXR, 1-deoxy-D-xylulose 5-phosphate reductoisomerase; GPPS, geranyldiphosphate synthase; FPPS, farnesyl pyrophosphate synthase; LS, linalool synthase; HK, hexokinase; ME, GDP-mannose-3′, 5′ -epimerase; IDH, L-idonate 5-dehydrogenase; PG, polygalacturonase; WRKY, WRKY DNA binding protein 4; MYB; ZE, zeaxanthin epoxidase; CYP, cytochrome P_450_

To validate the expression of putative genes in RNA-seq data, semi-quantitative and real-time PCR analyses were performed for selected transcripts belonging to terpene and tartaric acid pathways, transcription factor and hormone regulation. The comparative analysis revealed similar expression pattern as observed in transcriptome analysis (Fig. 9; Additional file 1: Figure S7).

## Conclusion

In this study, we have represented the comprehensive transcriptome assembly of high quality reads generated through Illumina pair end sequencing, into contigs and provided putative functional annotation of assembled transcripts of rose-scented geranium. Transcripts were identified for the enzymes involved in biosynthesis of terpene, ascorbic acid, tartaric acid and anacardic acid metabolites, predominant in rose-scented geranium*.* Transcriptome analysis notified presence of transcripts for idonate dehydrogenase that is involved in C4/C5 cleavage of ascorbate, suggesting existence of both C2/C3 and C4/C5 pathways of tartarate biosynthesis in rose-scented geranium*.* However, this needs to be further validated biochemically. Moreover, the orthologous genes related to hormones and transcription factors were identified. This transcriptome repository will serve as a platform to enrich our understanding about molecular mechanism of primary and secondary metabolic pathways of high importance, and metabolic engineering in rose-scented geranium. In addition, a large number of transcript based SSRs were identified, which could be potential molecular markers useful in functional genetic variation and marker-assisted breeding in rose-scented geranium.

## Additional files

Additional file 1: Figure S1. A phylogenetic tree generated on the basis of a plastid marker *trnL-F* in 57 *Pelargonium* species, and rose-scented geranium cv. Bournon. Figure S2, Categorization of rose-scented geranium transcriptome contigs based on gene ontology. Figure S3, MapMan visualized genes associated with primary metabolic biosynthesis pathways. Figure S4, MapMan visualized genes associated with secondary metabolic biosynthesis pathways. Figure S5, MapMan visualized genes associated with biotic and abiotic stress responses. Figure S6, PCR amplifications of selected putative genes for assembly validation. Figure S7, Semi quantitative PCR analysis of selected putative genes. (PDF 598 kb)
Additional file 2: Primers used in semi-quantitative PCR, real-time PCR and assembly validation. (XLSX 231 kb)
Additional file 3: Functional annotation of rose-scented geranium transcripts, putative annotation, and expression. Plant species contributing the annotated contigs in the top-scoring BLASTx hits against the NR protein database. (XLSX 548 kb)
Additional file 4: GO assignments of rose-scented geranium transcripts under biological processes, cellular components and molecular functions categories, number of contigs and percentage. (XLSX 11 kb)
Additional file 5: Details of rose-scented geranium transcripts matched with the *A. thaliana* protein database. (XLSX 2450 kb)
Additional file 6: Details of rose-scented geranium transcripts related with the enzymes involved in terpene biosynthesis. (XLSX 1631 kb)
Additional file 7: Details of rose-scented geranium transcripts related with the enzymes involved in ascorbic acid and tartaric acid biosynthesis. (XLSX 1501 kb)
Additional file 8: Details of rose-scented geranium transcripts related with the enzymes involved in anacardic acid biosynthesis. (XLSX 29 kb)
Additional file 9: Details of rose-scented geranium transcripts related with the transcription factors. (XLSX 23 kb)
Additional file 10: Details of rose-scented geranium transcripts related with hormones. (XLSX 18 kb)
Additional file 11: Details of SSRs identified in transcriptome data of rose-scented geranium. (XLSX 1273 kb)
