# Supplementary material for: De novo transcriptome analysis of rose-scented geranium provides insights into the metabolic specificity of terpene and tartaric acid biosynthesis
Source: BMC Genomics. 2017 Jan 13;18:74. doi: 10.1186/s12864-016-3437-0 (PMC5234130; doi:10.1186/s12864-016-3437-0)
Supplement: Additional file 1: — Figure S1. A phylogenetic tree generated on the basis of a plastid marker trnL-F in 57 Pelargonium species, and rose-scented geranium cv. Bournon. Figure S2, Categorization of rose-scented geranium transcriptome contigs based on gene ontology. Figure S3, MapMan visualized genes associated with primary metabolic biosynthesis pathways. Figure S4, MapMan visualized genes associated with secondary metabolic biosynthesis pathways. Figure S5, MapMan visualized genes associated with biotic and abiotic stress responses. Figure S6, PCR amplifications of selected putative genes for assembly validation. Figure S7, Semi quantitative PCR analysis of selected putative genes. (PDF 598 kb) [file 12864_2016_3437_MOESM1_ESM.pdf]

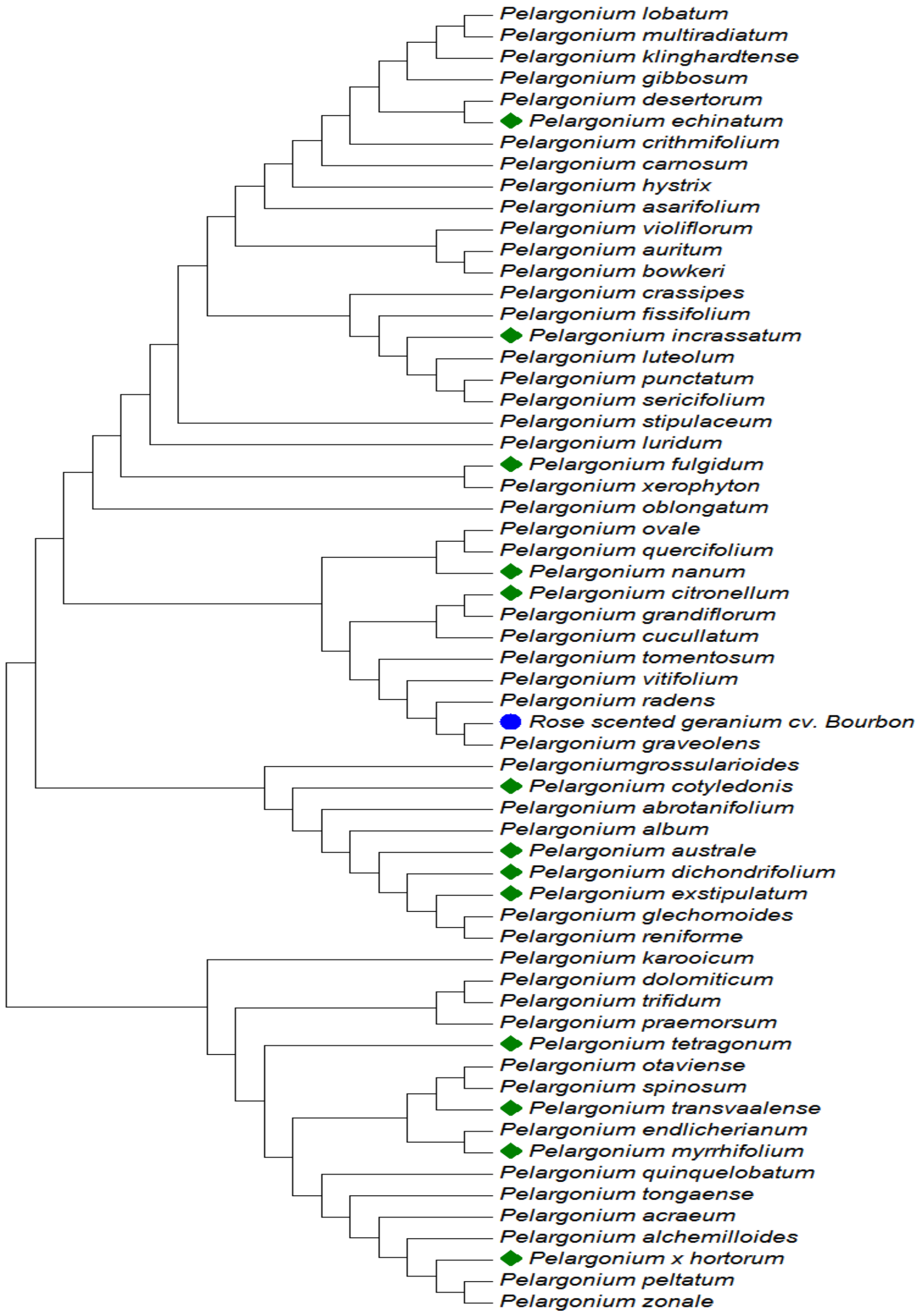

Figure S1 A phylogenetic tree generated on the basis of a plastid marker *trnL-F* in 57 *Pelargonium* species, and rose-scented geranium cv. Bournon. The molecular phylogenetic analysis was done by Maximum Likelihood method method based on the Tamura-Nei model [1], by using MEGA7 [2]. The nucleotide sequence information of *trnL-F* in 57 *Pelargonium* species was extracted from NCBI, and that of rose-scented geranium cv. Bournon (marked with blue bullet) was taken from the trancriptome assembly data. The 13 *Pelargonium* species, for which raw sequencing reads are available in SRA database, are marked with green bullets in the phylogenetic analysis to show its possible genetic distance from rose-scented geranium cv. Bourbon

Reference:

1. Tamura K and Nei M. Estimation of the number of nucleotide substitutions in the control region of mitochondrial DNA in humans and chimpanzees. Molecular Biology and Evolution 1993; 10:512-526.
2. Kumar S, Stecher G, and Tamura K. MEGA7: Molecular Evolutionary Genetics Analysis version 7.0 for bigger datasets. Molecular Biology and Evolution 2016; 33:1870-1874.

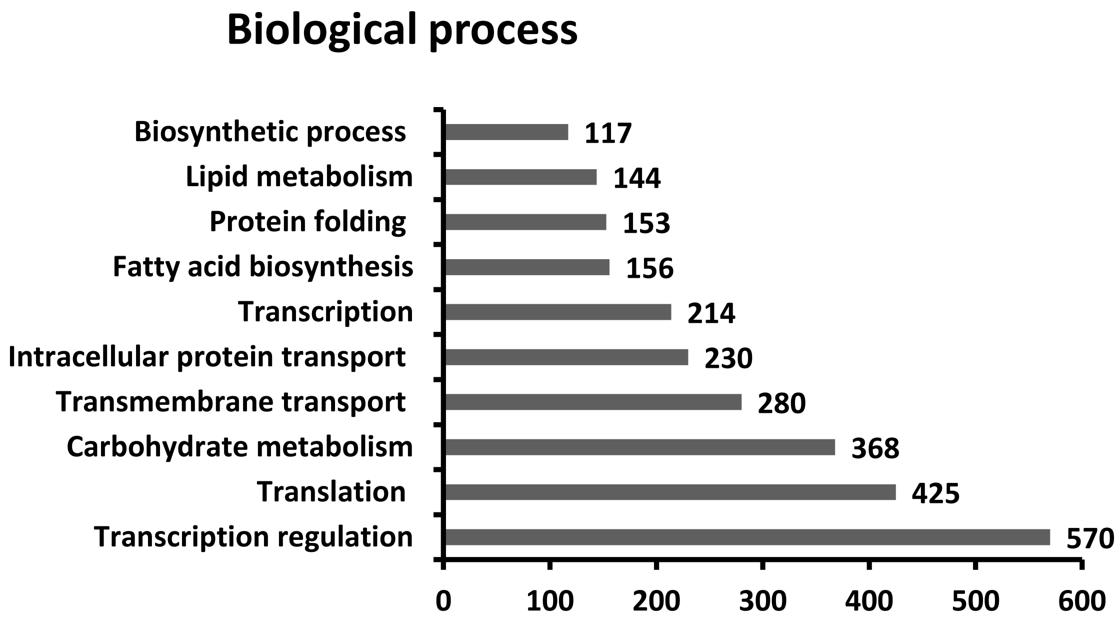

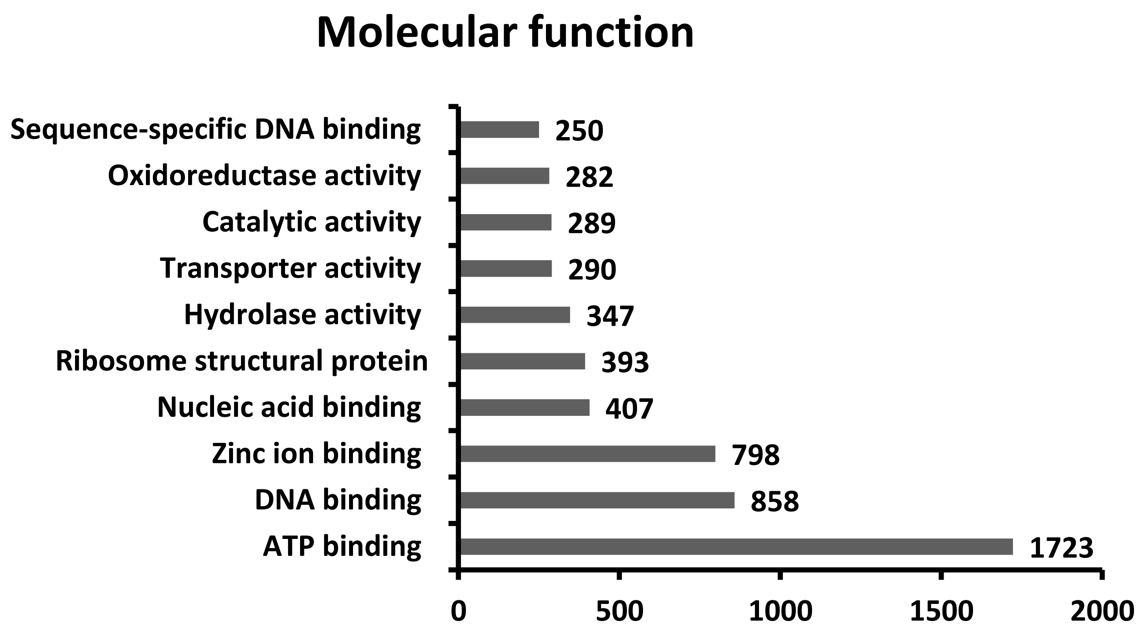

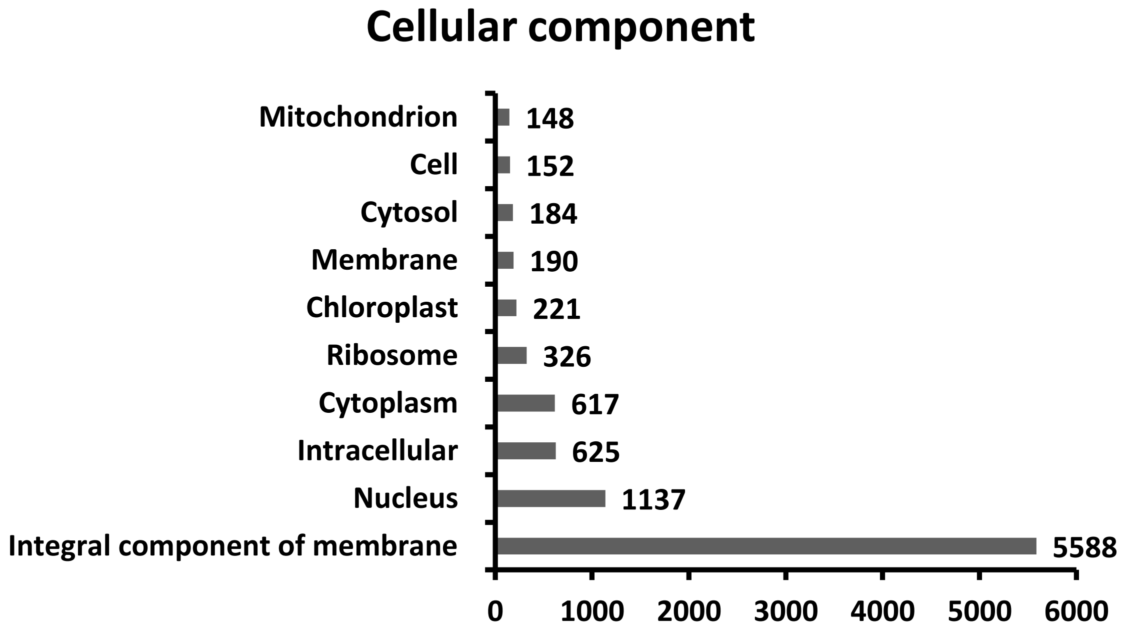

Figure S2 Categorization of rose-scented geranium transcriptome contigs based on gene ontology (biological processes, cellular components and molecular functions). Top 10 hits of each categories presented here.

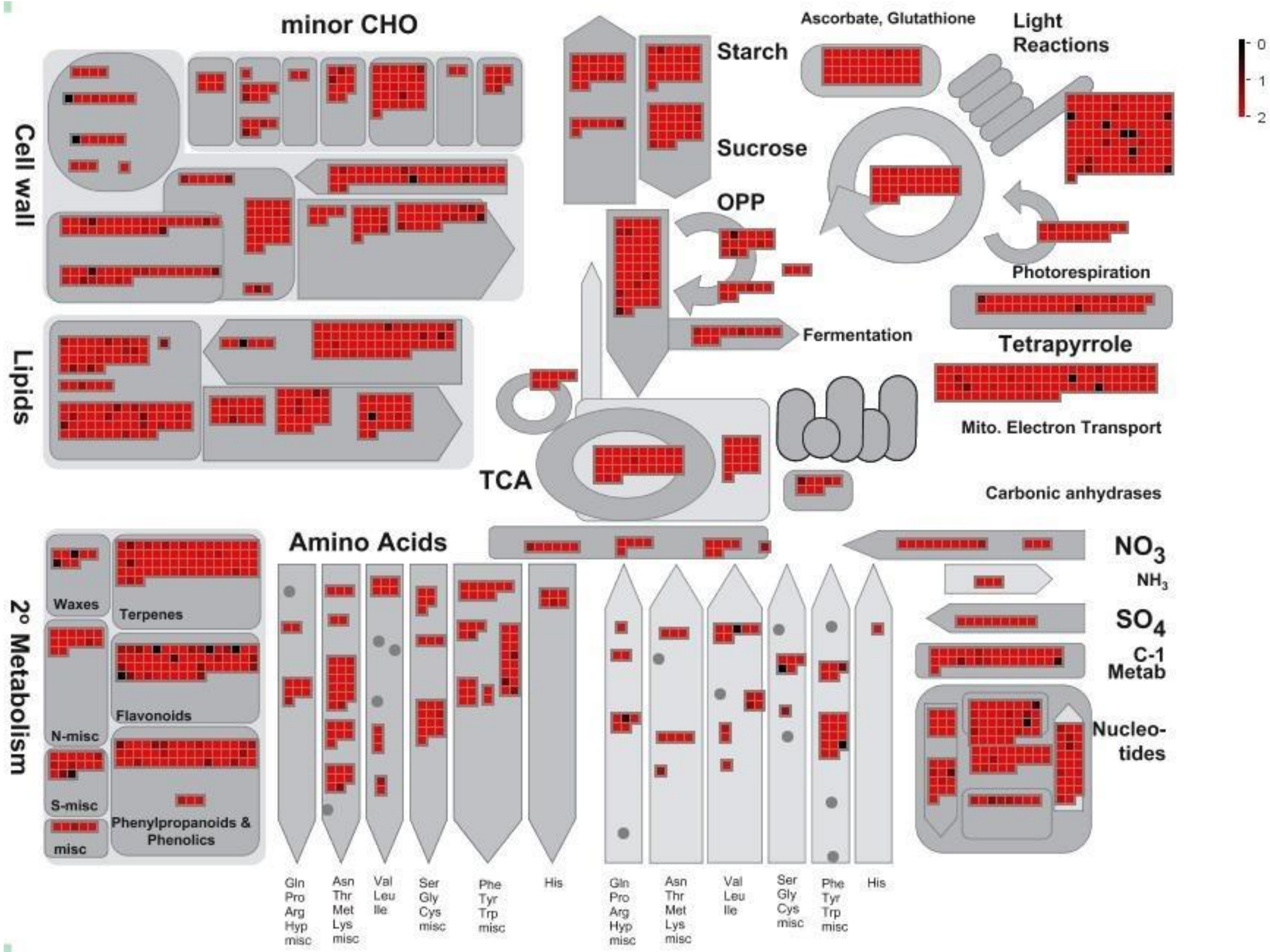

Figure S3 MapMan visualized genes associated with primary metabolic biosynthesis pathways.

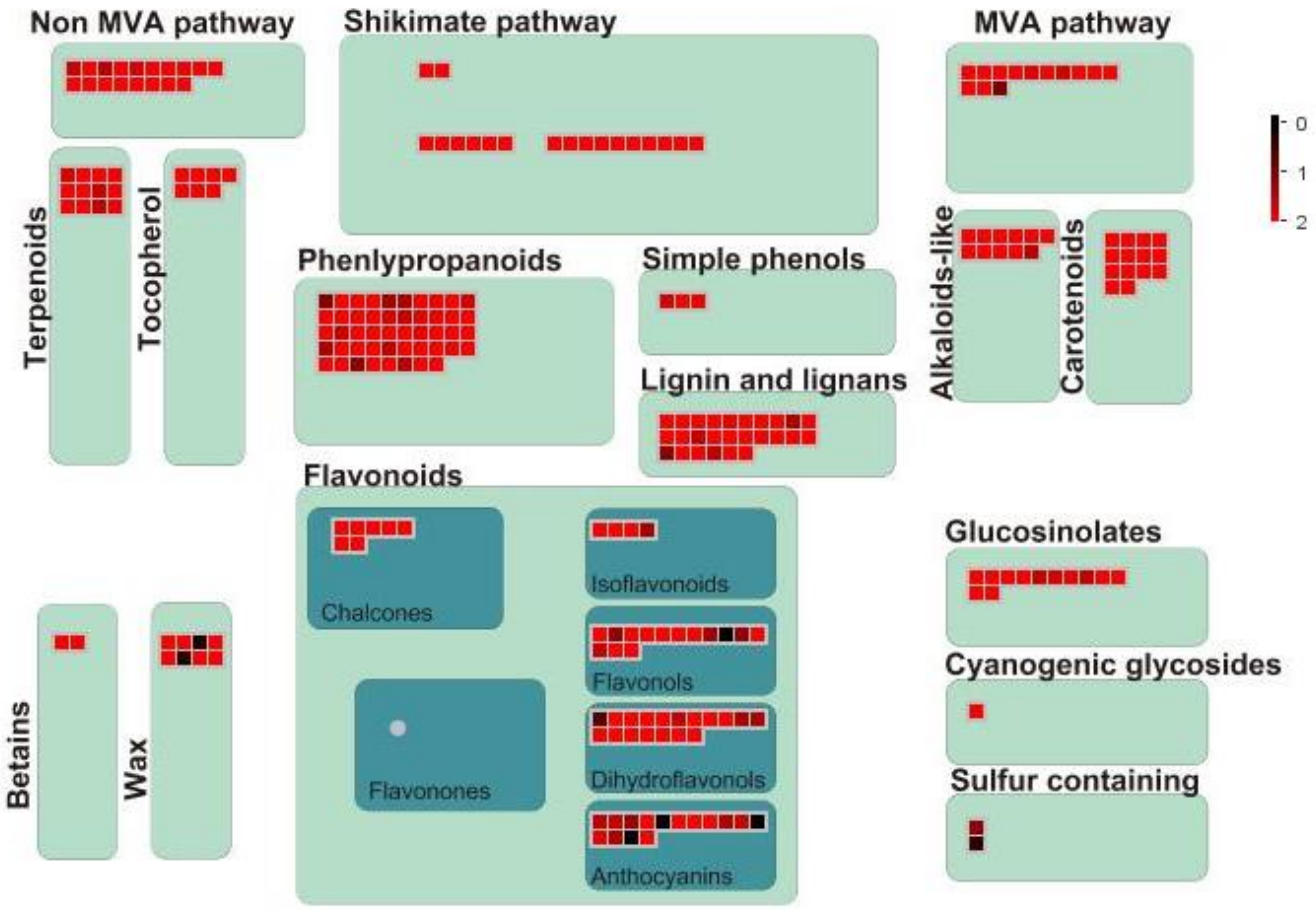

Figure S4 MapMan visualized genes associated with secondary metabolic biosynthesis pathways.

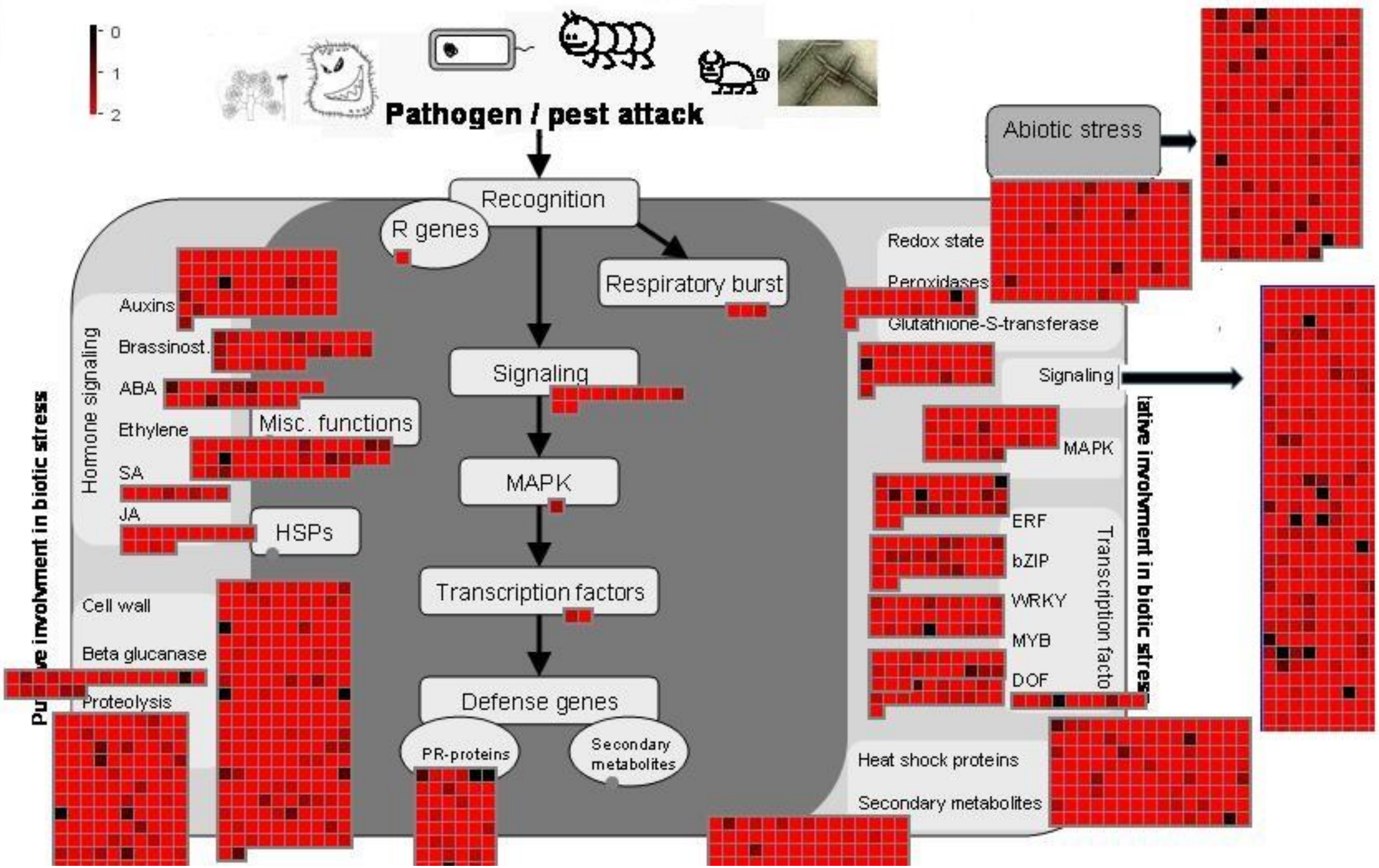

Figure S5 MapMan visualized genes associated with biotic and abiotic stress responses.

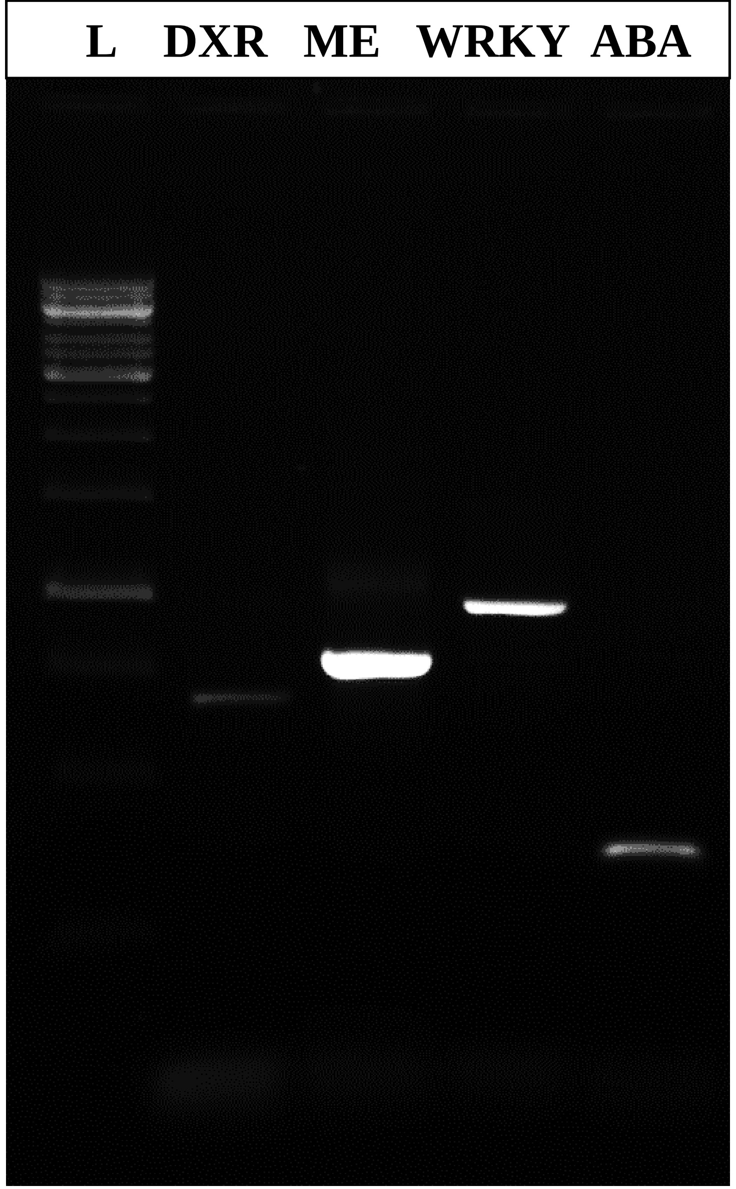

Figure S6 PCR amplifications of selected putative genes for assembly validation. Ladder; DXR- 1-Deoxy- D -xylulose 5-phosphate reductoisomerase; ME- GDP-mannose-3′,5′-epimerase; WRKY- WRKY transcription factor and ABA- Abscisic acid biosynthesis (Zeaxanthin epoxidase).

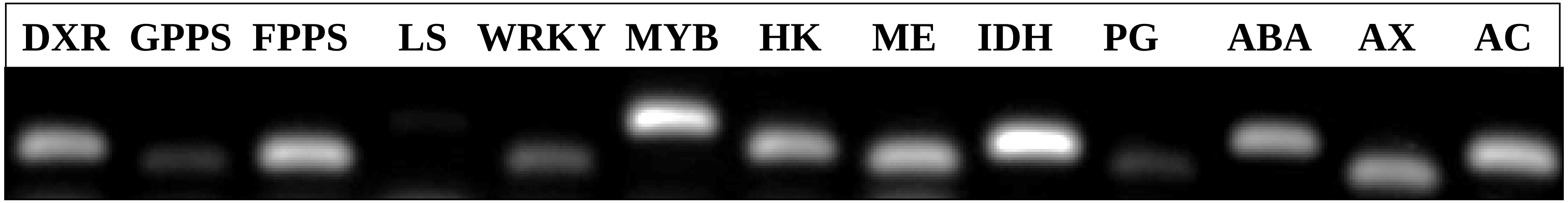

Figure S7 Semi-quantitative PCR analysis of selected putative genes. DXR- 1-Deoxy- D -xylulose 5-phosphate reductoisomerase; GPPS- Geranyl diphosphate synthase; FPPS- Farnesyl pyrophosphate synthase; LS- Linalool synthase; WRKY- WRKY transcription factor; MYB- MYB transcription factor; HK- Hexokinase; ME- GDP-mannose-3′,5′-epimerase; IDH- L-Idonate 5-dehydrogenase; PG- Polygalacturonase; ABA- Abscisic acid biosynthesis (Zeaxanthin epoxidase); AX- Auxin biosynthesis (Cytochrome P_{450}) and AC- Actin
